# Psychiatric, Neurophysical and Neurocognitive Sequelae of Post-Acute COVID-19 Syndrome: A Systematic Review

**DOI:** 10.1192/bjo.2022.256

**Published:** 2022-06-20

**Authors:** Arun Vincent, Oghenefejiro Ofovwe, Manfred Gschwandtner, Sukhi Shergill, Rafey Faruqui

**Affiliations:** 1 Kent & Medway NHS & Social Care Partnership Trust, Kent, United Kingdom; 2 Kent and Medway Medical School, Kent, United Kingdom

## Abstract

**Aims:**

COVID-19 causes cognitive, neurophysical and psychiatric sequalae that persist beyond the acute illness. These appear to be independent of the direct impact on respiratory function although the impact of multiorgan, especially brain pathology, may be a contributory factor – as may psycho-social effects of the disease. We performed a systematic review of literature to assess the sequelae of post-acute COVID-19 syndrome to better understand the need for dedicated interventions to improve functioning.

**Methods:**

We conducted a systematic review of reports included in MEDLINE, PsycINFO, and EMBASE. We searched for cohort studies exploring psychiatric and neuro-cognitive sequelae of post-acute COVID-19 in adults with a sample size of at least 100. The search was conducted on 4 February 2022. Findings are reported in line with Preferred Reporting Items for Systematic Reviews and Meta-Analyses (PRISMA). Two authors independently assessed the included studies’ methodological quality using The National Institute of Health (NIH) quality assessment tool for observational cohort and cross-sectional studies and all records were rated as good or fair.

**Results:**

Our search identified 66 records and 14 met protocol requirements. The studies varied in sample size ranging from 100 to 3762 participants. Time to follow-up ranged from 1–12 months. Main symptoms identified by a majority of the studies were; Fatigue (25% to 85%) and Sleep problems (20% to 79%). Psychiatric symptoms; Anxiety (19% to 56%), Depression (11% to 47%), PTSD (6% to 43%) and altered sense of reality (3% to 15%). Neuro-cognitive symptoms; Cognitive dysfunction (25% to 85%), brain fog (12% to 81%), memory problems (24% to 73%), concentration difficulties (25% to 54%), and attention deficit (27%).

Female sex, advanced age, pre-morbid asthma or COPD, increased disease severity, high BMI and new neurological complications during hospitalisation were some of the identified risk factors for persistent symptoms in post-acute COVID-19. One study identified male sex as a risk factor for moderate to severe PTSD. Current evidence suggests that symptoms decrease over time.

**Conclusion:**

There is clear evidence of neuro-physical, psychiatric and neurocognitive sequelae in post-acute COVID-19 syndrome. Differences in assessing and reporting findings makes it difficult to synthesize meaningful information. Identifying and formulating standardised assessments for outcome measures and reporting systems would be useful in future research. Further research into symptoms of post-acute COVID-19, to understand the pathophysiology will better enable us to raise public awareness, introduce preventative measures and incorporate appropriate treatment strategies for rehabilitation.

